# Brown Adipose Tissue—A Therapeutic Target in Obesity?

**DOI:** 10.3389/fphys.2018.01672

**Published:** 2018-11-23

**Authors:** Paul Trayhurn

**Affiliations:** ^1^Clore Laboratory, University of Buckingham, Buckingham, United Kingdom; ^2^Obesity Biology Unit, University of Liverpool, Liverpool, United Kingdom

**Keywords:** brown adipocyte, beige adipocyte, obesity, thermogenesis, uncoupling protein-1

Since brown adipose tissue (BAT) was first identified in the interscapular area of marmots by Gessner in 1551 (Smith and Horwitz, 1969), views on its functions have continued to evolve (Table 1). The initial proposition was that it was linked to hibernation, reflecting the fact that marmots are hibernating animals. This was followed sequentially by the view that BAT was part of the thymus (1670–1817), an endocrine gland active in the formation of blood (1817–63), a modified form of fat tissue providing a reservoir for food substances (1863–1902), and again as an endocrine gland (1902–61) (Afzelius, 1970). It was only in 1961 that the tissue was finally identified as an effecter of non-shivering thermogenesis (NST) and the “metabolic power” of brown adipocytes recognized (Smith and Horwitz, 1969).

**Table 1:** Evolution of perspectives on the physiological functions and characteristics of brown adipose tissue.

**Year**	**Function**
1551	Description by Gessner—role in hibernation
1670-1817	Part of the thymus
1817-1863	An endocrine gland—and active in the formation of blood
1863-1902	Modified form of fat tissue which serves as a reservoir for food substances
1902-1961	An endocrine gland once more
1961-	Thermogenic organ—non-shivering thermogenesis
1974-7	Elucidation of unique bioenergetic properties of brown fat mitochondria (proton leak)
1976-8	Discovery of UCP1
1978	Demonstration of quantitative importance to non-shivering thermogenesis in cold-acclimated rodents
1978/9-	Involved in energy balance (diet-induced thermogenesis) and obesity
2009-	Definitive identification of BAT in adult humans and its metabolic plasticity
2010/12	Discovery of “beige”/“brite” adipocytes
2011/12	Role in glucose disposal and triglyceride clearance – metabolic homeostasis

*Based partly on Afzelius (1970)*.

There were three situations where BAT was understood to be highly active—during arousal from hibernation, in the response to cold-exposure of certain adult mammals, and in cold-stressed mammalian neonates (Smith and Horwitz, 1969). The quantitative importance of BAT to NST was initially unclear. However, in the late 1970's it was clarified in the case of cold-acclimated rodents by employing radioactively microspheres to map regional blood flow, these studies demonstrating that brown fat may account for up to two-thirds of heat from NST (Foster and Frydman, 1978, 1979). The mechanism for heat generation in brown adipocytes was also being explored, and the uncoupling of oxidative phosphorylation through proton leakage across the inner mitochondrial membrane was established as the pathway by which thermogenesis occurs (Nicholls and Locke, 1984). This proton conductance pathway was shown to be dependent on, and regulated by, a cold-inducible mitochondrial uncoupling protein—UCP1 (Ricquier and Kader, 1976; Nicholls and Locke, 1984; Ricquier, 2017).

Since these pivotal advances, perspectives on the physiology of BAT have continued to evolve. Here, recent views on the role of the tissue are summarized and the potential implications for the treatment of obesity considered.

## Bat and nutritional energetics

A major new dimension emerged in the late 1970's with the establishment of a link between BAT and energy balance (Table 1). This resulted from two key observations, one of which was that rats exhibiting high rates of diet-induced thermogenesis on a cafeteria diet are characterized by an activation of BAT (Rothwell and Stock, 1979), including increased mitochondrial GDP binding indicative of an activation of the proton conductance pathway (Brooks et al., 1980). The other major observation was of decreased BAT activity, with a reduction in GDP binding, in obese (*ob/ob*) mice; this was proposed as a causative factor in the development of their obesity (Himms-Hagen and Desautels, 1978).

These pioneering observations resulted in a new paradigm in the etiology of obesity (Rothwell and Stock, 1981; Himms-Hagen, 1983, 1989; Trayhurn, 1989). A tissue and molecular basis for the reduced energy expenditure on facultative thermogenesis proposed as an important factor in the development of obesity had been identified. Abnormalities in BAT activity were subsequently reported in a range of animal obesities, including *db/db* mice and Zucker (*fa/fa*) rats, and in rodents with experimentally-induced obesity (Himms-Hagen, 1989; Trayhurn, 2017). The central role of BAT in nutritional energetics and body fat regulation was further evident from studies demonstrating changes in thermogenic activity in a range of physiological conditions—from fasting to lactation and photoperiod-induced seasonal changes in body weight (Trayhurn, 2017).

## Brown adipose tissue in man

A widely debated question in the 1980's was the extent to which studies on laboratory rodents were relevant to energetics and obesity in humans. This was effectively two questions—whether reduced expenditure on thermogenesis underpins the development of human obesity, and whether BAT is present in adults and thus the potential locus of impaired thermogenesis. The first question has been the source of continuing debate, but what seems clear is that thermogenesis is not a major component of expenditure in adult humans (Wijers et al., 2007; van Marken Lichtenbelt and Schrauwen, 2011)—certainly compared with small rodents such as mice in the cold where it accounts for up to two-thirds of total expenditure (Trayhurn and James, 1978; Trayhurn, 1979).

In the early 1980's, BAT was considered, essentially on histological criteria, to be absent in humans after the first years of life. This parallels precocial species such as goats and sheep in which BAT, while abundant at birth, rapidly loses UCP1 and thermogenic capacity over the first weeks of postnatal life being replaced by white fat (Casteilla et al., 1987; Trayhurn et al., 1993). Until 2009, the widely held view was that BAT (or thermogenic adipocytes) is absent from adult humans, despite anatomical studies identifying multi-locular adipocytes (Heaton, 1972), and more critically: (i) UCP1 being identified in adipose tissue depots of adults, including elderly subjects (Lean et al., 1986a), (ii) activation of BAT being demonstrated in phaeochromocytoma patients (Ricquier et al., 1982; Lean et al., 1986b), and (iii) *UCP1* gene expression in adipose tissue of adults, particularly in phaeochromocytoma (Bouillaud et al., 1988).

The lack of recognition that adults do contain active BAT was a key element in the decline of interest in the tissue by the 1990's. However, over the past decade several major developments have led to a renewed focus on BAT. First, studies from the late 2000's utilizing FDG-PET (fluorodeoxyglucose positron emission tomography), commonly employed to track tumor metastasis, have mapped areas of high glucose uptake which also contain UCP1, thereby firmly identifying active BAT in adults (Cypess et al., 2009; Virtanen et al., 2009; Moonen et al., 2019). Through such studies, increased BAT activity has been observed in response to multiple stimuli, including cold exposure (as in rodents), and administration of insulin and the β_3_-adrenoceptor agonist mirabregon (van Marken Lichtenbelt et al., 2009; Virtanen et al., 2009; Orava et al., 2011; Ouellet et al., 2012; Cypess Aaron et al., 2015). Importantly, the thermogenic activity of the tissue has been shown to decline with age and increasing BMI (Cypess et al., 2009; van Marken Lichtenbelt et al., 2009; Pfannenberg et al., 2010; Moonen et al., 2019).

## Recent developments in bat physiology

An important development over the past decade, although not on BAT as such, is the discovery of a third form of adipocyte—“brite” or “beige” (Petrovic et al., 2010; Wu et al., 2012; Carobbio et al., 2019). These fat cells contain UCP1, providing the potential for thermogenesis, and have some of the other molecular signatures of brown adipocytes (Petrovic et al., 2010; Wu et al., 2012). Beige adipocytes are found primarily within WAT depots, and are recruited particularly during cold-exposure and following β-adrenergic stimulation resulting in the “browning” of white fat (Carobbio et al., 2019).

A further advance comes from evidence suggesting that BAT plays an important role in metabolic homeostasis, the tissue being a major site of glucose disposal, insulin action and triglyceride clearance (Bartelt et al., 2011; Stanford et al., 2013; Bartelt and Heeren, 2014). A high capacity for glucose uptake, which can be stimulated by insulin and cold, is evident from FDG-PET studies. Roles for BAT in glucose homeostasis and triglyceride clearance have led to the proposition that reduced activity in the tissue may underlie the metabolic syndrome (Nedergaard et al., 2011; Bartelt and Heeren, 2014; Moonen et al., 2019).

An additional dimension is the function of BAT as a secretory organ. Apart from the release of fatty acids, brown adipocytes synthesize and secrete multiple peptide/protein factors, these “brown adipokines” paralleling the adipokines of white adipocytes (Villarroya et al., 2013, 2017). While the adipokinome of white fat cells numbers several hundred entities (Trayhurn, 2013), it is unclear whether the secretome from brown adipocytes is as extensive. The brown adipokinome includes classical adipokines such as leptin and IL-6 (Villarroya et al., 2013, 2017), but brown adipocytes are unlikely to be an important source of these factors.

## Bat as a therapeutic target—is it realistic?

A major driver behind the renewed interest in BAT relates to its potential as a therapeutic target to treat obesity. This reflects the revival of a concept current in the 1980's, but it is now based on the firm acceptance that the tissue is present, thermogenically active and exhibits plasticity in adults. The ability to recruit beige adipocytes within WAT has further added to the focus on BAT (Petrovic et al., 2010; Wu et al., 2012; Jespersen Naja et al., 2013; Sacks et al., 2013), or more strictly on UCP1-dependent adipocyte thermogenesis, as a therapeutic route in obesity. But how realistic is this?

### The case in favor

There are substantive points in favor of BAT as a target—which in principal can be through increasing the thermogenic activity/capacity of pre-existing brown adipocytes, by recruitment of new brown adipocytes, or a combination of the two. Additionally, there is the potential to recruit beige cells through “browning.” BAT has the conceptual attraction of being a clearly defined target with a precise molecular end-point in the amount and activity of UCP1. It also centres on one specific element of energy balance—adaptive energy expenditure—so augmentation is a logical approach. Furthermore, as well as increasing expenditure it has the potential of improving metabolic homeostasis through its role in glucose utilization and lipid clearance (Nedergaard and Cannon, 2010; Bartelt and Heeren, 2014; Moonen et al., 2019).

### The case against

There are, nevertheless, also substantive arguments against BAT as a realistic target. A key requirement is that the stimulation of energy expenditure through BAT does not lead to a compensatory increase in energy intake, which would be counter-productive. It is uncertain whether compensation would occur under normal circumstances; however, increased intake is very evident in rodents in the cold, with for example mice consuming three times more food at 4°C than at thermoneutrality (Trayhurn, 1981), suggesting that increased BAT activity is linked to higher intake.

A critical issue is the quantitative importance of BAT thermogenesis to energy expenditure in adults. Clearly, if the contribution is minimal then it is a less obvious target for modulating energy balance—though a small, but sustained, imbalance between expenditure and intake would be effective. Estimates of the importance of BAT in energy expenditure range from 1 to 5% of RMR, but these are in effect basal values and it is argued that with stimulation the value will be higher at up to 16% of RMR (van der Lans et al., 2013; Moonen et al., 2019)–this can be viewed as part of the “case in favor.”

A number of approaches to increasing the thermogenic activity and capacity of BAT have been proposed. The initial route was to design selective β_3_-agonists, reflecting the centrality of the β_3_-adrenoceptor in the regulation of thermogenesis, and several were developed (Arch, 2002). However, although the first generation compounds were effective in stimulating expenditure and reversing obesity in rodents, their efficacy and potency in humans is poor due to a combination of factors including sequence differences between the human and rodent β_3_-adrenoceptor (Arch, 2002). Nevertheless, selective β_3_-agonists continue to be of interest with mirabegron having recently been shown to stimulate RMR and glucose uptake by BAT (Cypess Aaron et al., 2015). Alternative strategies have been proposed and these include central stimulation of the sympathetic innervation to BAT, brown fat transplantation and stem cell therapy (Gunawardana and Piston, 2012; Nishio et al., 2012; Guénantin et al., 2017; Carobbio et al., 2019). While potentially feasible, it is difficult to regard transplantation and stem cell therapy as practical approaches to obesity treatment.

Recruitment of beige cells would augment the number of thermogenic adipocytes, and multiple factors can facilitate this process (Bartelt and Heeren, 2014; Nedergaard and Cannon, 2014; Carobbio et al., 2019). There are, however, potential constraints on whether beige adipocytes may provide significant amounts of heat—apart from any intracellular limitation. To make a significant contribution to NST, close proximity to an extensive vascular network would be expected, both to rapidly dissipate the heat generated and to provide oxygen and other nutrients to fuel high rates of thermogenesis. Furthermore, thermogenesis requires noradrenergic stimulation, and the recruitment of beige adipocytes needs to be accompanied by local growth of the sympathetic innervation. A report that alternatively activated macrophages stimulate thermogenesis in BAT through the secretion of catecholamines (Nguyen et al., 2011) has not been supported subsequently (Fischer et al., 2017); however, the hormone meterorin-like promotes browning through M2 macrophage activation (Rao et al., 2014).

The final reservation to treating obesity through the stimulation of BAT thermogenesis lies in the potential cardiovascular risk. While this may be small, and needs to be balanced against the risks associated with obesity itself, regulatory agencies are likely to be ultra-cautious given the readiness with which previous anti-obesity drugs have been withdrawn following evidence of adverse effects.

## Concluding comments

Our understanding of the functions of BAT has continued to evolve and it is evident that the tissue has multiple actions beyond a core thermoregulatory role. Recognition of its involvement in nutritional energetics and the etiology of obesity in the late 1970's transformed perspectives on the tissue. Over the past decade there has been a renaissance of interest in BAT, following clear evidence of its presence and metabolic plasticity in adult humans. There is currently considerable focus on BAT as a therapeutic target in the treatment of obesity and its associated metabolic disorders, aided by the identification of beige adipocytes and the phenomenon of browning of WAT. However, it is suggested that in totality there are formidable barriers to this approach and that the stimulation and recruitment of thermogenic adipocytes is unlikely to provide a realistic anti-obesity/anti-metabolic syndrome strategy in the near-future.

## Author contributions

The author confirms being the sole contributor of this work and has approved it for publication.

### Conflict of interest statement

The author declares that the research was conducted in the absence of any commercial or financial relationships that could be construed as a potential conflict of interest.
